# Quantitative Analysis of Sphingomyelin by High-Performance Liquid Chromatography after Enzymatic Hydrolysis

**DOI:** 10.1155/2012/396218

**Published:** 2012-08-05

**Authors:** Seunghyun Lee, Youn-Sun Lee, Kyeong-Mi Choi, Kwang-Sik Yoo, Dong-Mi Sin, Wonkyun Kim, Yong-Moon Lee, Jin-Tae Hong, Yeo-Pyo Yun, Hwan-Soo Yoo

**Affiliations:** College of Pharmacy and Medical Research Center, Chungbuk National University, Cheongju 361-763, Republic of Korea

## Abstract

Sphingomyelin is the most abundant sphingolipid in mammalian cells and is mostly present in the plasma membrane. A new analytical method using high-performance liquid chromatography (HPLC) was developed to quantify sphingomyelin in mouse plasma and tissues, 3T3-L1 cells, rat aortic smooth muscle cells, and HT-29 cells. Sphingomyelin and dihydrosphingomyelin, an internal standard, were separated by high-performance thin-layer chromatography and simultaneously hydrolyzed with sphingolipid ceramide *N*-deacylase and sphingomyelinase to release sphingosine and dihydrosphingosine, respectively. Sphingomyelin content was measured by HPLC following *o*-phthalaldehyde derivatization. Sphingomyelin concentrations in 3T3-L1 cells, rat aortic smooth muscle cells, and HT-29 cells were 60.10 ± 0.24, 62.69 ± 0.08, and 58.38 ± 0.37 pmol/**μ**g protein, respectively, whereas those in brain, kidney, and liver of ICR mice were 55.60 ± 0.43, 43.75 ± 0.21, and 22.26 ± 0.14 pmol/**μ**g protein. The sphingomyelin concentration in mouse plasma was 407.40 ± 0.31 **μ**M. The limits of detection and quantification for sphingomyelin were 5 and 20 pmol, respectively, in the HPLC analysis with fluorescence detection. This sensitivity was sufficient for analyzing sphingomyelin in biological samples. In conclusion, this analytical method is a sensitive and specific technique for quantifying sphingomyelin and was successfully applied to diverse biological samples with excellent reproducibility.

## 1. Introduction

Sphingomyelin is the most abundant sphingolipid in mammalian cells and is mostly present in the plasma membrane. Sphingomyelin has a long-chain sphingosine base with an amide-linked fatty acyl chain and a phosphorylcholine head group at the 1 position of the hydrocarbon backbone. Sphingomyelin is associated with cell growth, differentiation, and apoptosis. Sphingomyelin hydrolysis results in the generation of ceramide in response to a variety of tumor necrosis factor receptor ligands, anticancer drugs, oxidants, and other cellular stresses [1].

Several analytical methods have been developed to quantify sphingomyelin, including high-performance thin-layer chromatography (HPTLC), gas chromatography (GC), nuclear magnetic resonance (NMR), and mass spectrometry (MS). HPTLC plates with efficient, fine particle layers produce very high resolution to separate sphingomyelin from other sphingolipids [2]. However, HPTLC alone is insufficient to quantify sphingomyelin. Sphingomyelin is hydrolyzed by sphingomyelinase (SMase) to liberate ceramide and then derivatized for the GC analysis, because sphingomyelin is not volatile [3]. However, the sensitivity and quality of analyses vary because of the high column temperature. Plasma sphingomyelin concentrations from different animal species have also been determined by ^31^P NMR analysis [4]. However, the NMR method to quantify sphingomyelin is limited for applying to diverse samples. Different sphingomyelin structures have been identified by mass spectra using delayed extraction matrix-assisted laser desorption/ionization-time-of-flight mass spectrometry [5]. MS analyses can quantify the molecular species of sphingomyelin with high sensitivity. However, the sensitivity can be dependent on the mode of ionization and may vary with molecular species.

**Figure 1:**
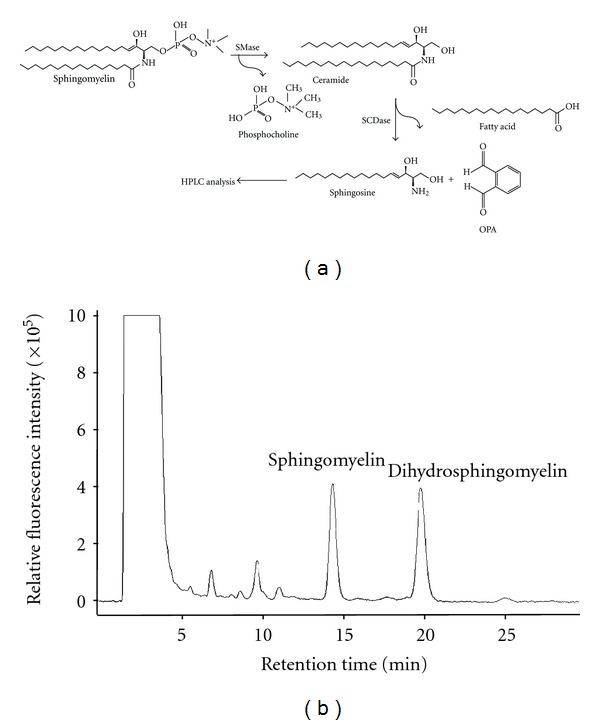
Sphingomyelin quantification procedure and the HPLC chromatogram. (a) Sphingomyelin was simultaneously hydrolyzed with sphingomyelinase (SMase) and sphingolipid ceramide *N*-deacylase (SCDase), and the released sphingosine was derivatized with OPA followed by HPLC analysis. (b) Sphingomyelin was separated from lipid extracts of ICR mouse kidney tissue by high-performance thin-layer chromatography (HPTLC), simultaneously hydrolyzed with SMase and SCDase, and analyzed by HPLC following OPA derivatization. The peaks representing sphingomyelin and dihydrosphingomyelin (internal standard) occurred at 14.4 and 19.8 min, respectively.

In a previous study, an analytical method to quantify ceramide utilized HPLC, following HPTLC purification, sphingolipid ceramide *N*-deacylase (SCDase) deacylation, and *o*-phthalaldehyde (OPA) fluorescence derivatization [6]. In this study the modified analytical method was applied to determine sphingomyelin concentrations in biological samples. The present analytical procedure comprised HPTLC separation, sphingomyelin hydrolysis using SCDase and SMase, OPA fluorescence derivatization, and HPLC analysis (Figure 1(a)). Additionally, spiking dihydrosphingomyelin into biological samples as an internal standard compensates for variations due to multistep procedures and enables the actual amount of endogenous sphingomyelin to be measured. The present method was more specific and reproducible than previously published methods for determining total content of sphingomyelin and could be applied to a diversity of biological samples. 

## 2. Materials and Methods

### 2.1. Materials

Sphingomyelin, dihydrosphingomyelin, ceramide, sphingosine-1-phosphate, and ceramide-1-phosphate were purchased from Avanti Polar Lipids, Inc. (Alabaster, AL, USA). SCDase from *Pseudomonas* sp. was obtained from Takara Bio. Inc. (Tokyo, Japan), and SMase from *Staphylococcus aureus* was obtained from Sigma (St. Louis, MO, USA). HPTLC silica-gel plate, chloroform, and HPLC grade methanol were purchased from Merck (Darmstadt, Germany). OPA was obtained from Molecular Probes, Inc. (Eugene, OR, USA). Myriocin was purchased from Biomol Research, Inc. (Plymouth Meeting, PA, USA). All other reagents were of the highest purity available.

### 2.2. Cell Culture

3T3-L1 cells were cultured in Dulbecco's Modified Eagle Medium (DMEM) containing 10% bovine calf serum at 37°C in a 5% CO_2_ atmosphere until the cells were fully confluent. Rat aortic smooth muscle cells (RASMCs) were cultured in DMEM supplemented with 10% fetal bovine serum (FBS), 100 units/mL penicillin, 100 *μ*g/mL streptomycin, 8 mM HEPES, and 2 mM L-glutamine at 37°C in a 5% CO_2_ atmosphere. HT-29 cells were cultured in DMEM supplemented with 10% FBS, 100 units/mL penicillin, at 37°C in a 5% CO_2_ atmosphere.

### 2.3. Animals

This study was approved by the ethical committee of animal experiments at Chungbuk National University, and all animal procedures were performed in accordance with the Public Health Service policy. ICR mice (age, 7-8 weeks; weight, 20 g, *n* = 10) were purchased from Daehan Biolink (Eumsung, Republic of Korea) and acclimated for 1 week in the animal facility (22°C, 12 h light/dark cycle). The animals had free access to drinking water and a commercial pellet diet obtained from Samyang Co. (Wonju, Republic of Korea). Blood was collected, and organs (brain, kidney, and liver) were removed for sphingolipid analysis.

### 2.4. Lipid Extraction and Sphingomyelin Separation

Biological samples for lipid extraction included cultured cells and tissues and plasma from mice. Total lipids were extracted with 1 mL chloroform/methanol (1 : 2, v/v) at 37°C for 1 h following the addition of dihydrosphingomyelin as an internal standard. The extract was centrifuged at 15,000 ×g for 10 min, and the supernatant layer was transferred to a polypropylene tubes and dried in a Speed-Vac concentrator (Vision Scientific Co., Daejeon, Republic of Korea). The dried residue was dissolved in 30 *μ*L of chloroform/methanol (1 : 2, v/v) and spotted on an HPTLC silica-gel plate. The plate was developed in chloroform/methanol/2 M aqueous ammonia (65 : 25 : 4, v/v/v). Sphingomyelin standard lanes were cut from the sample lanes of the HPTLC plate, and the sphingolipid standard was visualized by dipping the plate in 10% sulfuric acid and drying at 170°C. The areas in the sample lane with the same Rf values as the visualized sphingomyelin standard band were scraped off, and both sphingomyelin and dihydrosphingomyelin were eluted with 1 mL chloroform/methanol (1 : 2, v/v). The eluates were transferred to polypropylene tubes and dried. 

### 2.5. Enzymatic Hydrolysis

The sphingomyelin residue was mixed with reaction buffer containing 25 mM Tris-HCl buffer, pH 7.5, 1% sodium cholate, 15% fatty acid-free BSA, 150 *μ*units SCDase, and 20 *μ*units SMase. Sphingomyelin was simultaneously hydrolyzed with SCDase and SMase at 37°C for 12 h to release sphingosine. BSA was precipitated in the reaction buffer by adding ethanol and removed by centrifugation, and the supernatant was dried. 

### 2.6. HPLC Analysis

The sphingolipid extracts were dissolved in 120 *μ*L methanol, mixed with 15 *μ*L OPA reagent (50 mg OPA, 1 mL ethanol, 200 *μ*L *β*-mercaptoethanol, and 50 mL 3% (w/v) boric acid buffer, pH 10.5), and incubated at room temperature for 30 min for derivatization. The HPLC analysis was performed using a Shimadzu model LC-10AT pump, a SIL-10AXL autosampler system (Tokyo, Japan), and an analytical Radial-Pak cartridge (Waters Associates, Inc., Milford, MA, USA) packed with Nova-Pak C18 reversed-phase column (4 *μ*m, 100 mm × 8 mm). The isocratic mobile phase composition of methanol/distilled water/triethylamine (92 : 8 : 0.1, v/v/v) and a flow rate of 1.0 mL/min were accurately regulated by the HPLC system controller (Shimadzu SCL-10A). The Shimadzu RF-10XL fluorescence detector was set at an excitation wavelength of 340 nm and an emission wavelength of 455 nm. 

### 2.7. Protein Assay

Cell pellets and tissue homogenates were ultrasonicated in 1 mL PBS, and a part of the lysates was mixed with Pierce BCA reagents (Rockford, IL, USA) and incubated for 30 min. Protein content was quantified with a Molecular Devices ELISA reader (Sunnyvale, CA, USA) at 562 nm based on a bovine serum albumin (BSA) standard curve.

### 2.8. Statistical Analysis

All values are expressed as the mean ± standard deviation. One-way analysis of variance was used followed by the Newman-Keuls multiple comparison test. *P* values < 0.05 were considered statistically significant. 

## 3. Results

### 3.1. Establishment of Sphingomyelin Analysis

Sphingomyelin is the most abundant sphingolipid in mammalian cells and is ubiquitous lipid, mostly present in plasma membranes. Sphingomyelin is produced from *de novo* synthesis or by turnover pathway. Sphingomyelin can be metabolized to ceramide by SMase and is involved in apoptosis, proliferation, and senescence [7–11]. Several methods using HPTLC, GC, NMR, and MS have been developed to quantify sphingomyelin. However, HPTLC alone is insufficient to quantify sphingomyelin. Sphingomyelin is not volatile enough for GC analysis. The sensitivity and quality of a GC analysis generally vary because of high column temperature. The NMR method for sphingomyelin analysis appears to have a limitation to apply to a diversity of biological samples. The sensitivity of MS analysis is dependent on the mode of ionization and may vary with molecular species. 

In this study, the ceramide analytical method was modified to determine sphingomyelin concentrations in a diversity of tissues and cultured cells [12]. The present analytical procedure included HPTLC separation, sphingomyelin hydrolysis using SCDase and SMase, OPA fluorescence derivatization, and HPLC analysis (Figure 1(a)). The sphingomyelin and dihydrosphingomyelin peaks on the HPLC chromatogram had retention times of 14.4 and 19.8 min, respectively (Figure 1(b)). Peaks representing sphingomyelin and dihydrosphingomyelin on HPLC chromatogram are sphingosine and dihydrosphingosine, respectively, which can be analyzed by HPLC after OPA fluorescence derivatization.

This HPLC method is more specific and reproducible than previously published methods to measure sphingomyelin content. Sphingomyelin and dihydrosphingomyelin of samples after HPTLC separation were specifically hydrolyzed with SCDase and SMase to produce sphingosine and dihydrosphingosine, respectively, which can be analyzed by HPLC (Figure 1(a)). For the aspect of specificity, SMase cleaves the phosphodiester bond of sphingomyelin yielding ceramide and phosphocholine, and SCDase hydrolyzes the *N*-acyl linkage between fatty acids and sphingoid bases in ceramide.

The introduction of a proper internal standard, dihydrosphingomyelin, into the biological samples improved precision and enhanced the reproducibility of sphingomyelin quantification. The present analytical method proceeded in 1.5 mL polypropylene tubes. Large-scale analyses using glass tubes are inconvenient and time-consuming and might limit the number of samples. The increased sensitivity of our method allowed for small-scale analysis that could be applied to a large number of samples.

When the lipid extract of mouse brain tissue was analyzed without the HPTLC procedure, the HPLC chromatogram showed many nonspecific peaks that interfered with the authentic sphingolipid peaks, although OPA fluorescence detection is specific for the amino groups of analytes (Figure 3(a)). Separating sphingomyelin from the lipid extract by HPTLC provided an HPLC chromatogram with a clean background, and sphingomyelin could be quantified (Figure 3(b)).

### 3.2. Sphingomyelin Separation by HPTLC

**Figure 2:**
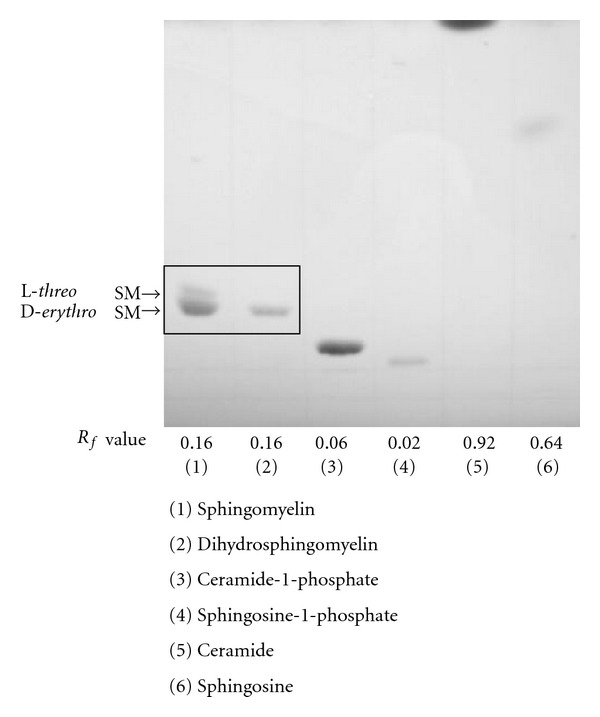
HPTLC chromatogram of sphingolipid standards. Sphingomyelin, dihydrosphingomyelin, ceramide-1-phosphate, sphingosine-1-phosphate, ceramide, and sphingosine standards were spotted on the HPTLC plate, developed in chloroform/methanol/2 M aqueous ammonia (65 : 25 : 4, v/v/v) for 1 h, and visualized with 10% sulfuric acid.

Possible contamination with ceramide-1-phosphate, sphingosine-1-phosphate, ceramide, and sphingosine was excluded by the HPTLC separation (Figure 2). An HPTLC plate, loaded with sphingomyelin standard in the end lanes and samples in the inside lanes, was developed with a mixture of chloroform/methanol/2 M aqueous ammonia (65 : 25 : 4, v/v/v). The sphingomyelin standard lanes were cut from the HPTLC plate, and the sphingomyelin standard band was visualized with sulfuric acid. Sphingomyelin appeared to be the same band as the dihydrosphingomyelin standard on the HPTLC plate with an Rf value of 0.16. This HPTLC solvent development system allowed the sphingomyelin band to be separated from the other sphingolipids. The Rf values for sphingomyelin, ceramide-1-phosphate, sphingosine-1-phosphate, ceramide, and sphingosine after 1 h development were 0.16, 0.06, 0.02, 0.92, and 0.64, respectively.

### 3.3. Optimization of Enzymatic Hydrolysis Reaction to Release Sphingosine

The present analytical method for sphingomyelin includes sphingomyelin hydrolysis using SMase and SCDase. SMase cleaves the phosphodiester bond of sphingomyelin yielding ceramide and phosphocholine, and SCDase hydrolyzes the *N*-acyl linkage between fatty acids and sphingoid bases in either sphingomyelin or ceramide. Sphingomyelin was hydrolyzed simultaneously with SCDase and SMase for 12 h to release sphingosine. Sphingomyelin hydrolysis occurred efficiently by fatty acid-free BSA, because BSA enhances sphingomyelin solubility. The degree of sphingomyelin hydrolysis increased with 0–15% BSA concentrations and was maximum at 15% BSA (Figure 4(a)). The dihydrosphingomyelin enzymatic reaction pattern was similar to that for sphingomyelin (Figure 4(b)). Sphingomyelin and dihydrosphingomyelin standards were incubated for varying periods of time (0.5, 1, 2, 4, 8, 12, 16, 20, and 24 h), followed by HPLC analysis. The optimal incubation time for sphingomyelin and dihydrosphingomyelin hydrolysis was 12 h (Figure 5(a)). Approximately 50% of the sphingolipid hydrolysis occurred during the first 30 min, and the enzymatic reaction began to decrease after 12 h. Sphingomyelin and dihydrosphingomyelin standards (10–1,000 pmol) were for 12 h, followed by HPLC analysis. Enzymatic hydrolysis was linear in the range of 10–1000 pmol with an *R* ^2^ value of 0.9991 between sphingomyelin concentration and peak area (Figure 5(b)). The linearity of dihydrosphingomyelin hydrolysis was similar to that for sphingomyelin with an *R* ^2^ value of 0.9973. The optimum amounts of SMase and SCDase were 20 and 150 *μ*units, respectively, for enzymatic hydrolysis of sphingomyelin (Figure 5(c)). 

### 3.4. Precision and Reproducibility of the Analytic Method

Known amounts (100–1,000 pmol) of sphingomyelin standard were added to determine the intra- and inter-day coefficients of variation (CV) for the sphingomyelin standard (Table 1). The intra-day CV was 0.71–2.08% (*n* = 10), and that for the inter-day analysis was 0.70–2.22% (*n* = 10). 

The limits of detection (*S*/*N* = 3) and quantification (*S*/*N* = 10) for sphingomyelin in the HPLC analysis with fluorescence detection were 5 and 20 pmol, respectively. This sensitivity was sufficient for analyzing sphingomyelin in small biological samples (5 *μ*L for mouse plasma).

In intra-day analysis, the amounts of sphingomyelin in mouse plasma were linearly related with the volumes of samples (Table 2). Mean CV and accuracy values of mouse plasma were 2.56 and 99.46%, respectively. Sphingomyelin contents, accuracy, and CV of mouse liver and 3T3-L1 cells showed the similar profile with those of mouse plasma. Mean CV and accuracy values of sphingomyelin contents from inter-day analysis of mouse plasma were 2.48 and 99.36%, respectively.

### 3.5. Determination of Sphingomyelin Concentrations in Biological Samples

The levels of sphingomyelin in mouse plasma and tissues and cultured cells were determined in 1.5 mL polypropylene tubes (Table 3). Sphingomyelin concentration in mouse plasma is about 407 *μ*M. The contents of cellular sphingomyelin in brain, kidney, and liver of ICR mice were 55.60 ± 0.43, 43.75 ± 0.21, and 22.26 ± 0.14 pmol/*μ*g protein, respectively. The effect of ageing on sphingomyelin content of rat brain and liver mitochondria has been studied [13, 14]. Levels of sphingomyelin in the brain from young adult (8–10 weeks) and old (18–24 months) rats were 27.45 ± 1.11 and 47.72 ± 3.76 *μ*g/mg protein, respectively, when the analysis was performed with the phosphorous assay after separation by column chromatography. If N16:0 sphingomyelin is assumed to be the major species, the sphingomyelin contents in the brain from young adult and old rats were calculated as 39.05 ± 1.58 and 67.88 ± 5.35 pmol/*μ*g protein, respectively. Thus, the sphingomyelin level in brain tissue from rats according to the phosphorous assay appeared to be similar to that from mice using the present method. In this study, sphingomyelin concentrations in 3T3-L1 cells, HT-29 cells, and RASMCs were 60.10 ± 0.24, 58.38 ± 0.37, and 62.69 ± 0.08 pmol/*μ*g protein, respectively. The amounts of sphingomyelin in influenza viruses derived from normal and Niemann-Pick disease type C F92-99 fibroblasts are approximately 105 and 120 pmol/*μ*g protein, respectively, by electrospray ionization MS [15]. 

Myriocin, an inhibitor of serine palmitoyltransferase in *de novo *sphingolipid synthesis, decreased sphingomyelin contents in 3T3-L1 preadipocytes for 1–72 h in a time-dependent manner, whereas sphingolipid content in control cells increased (Figure 6). Sphingomyelin concentration began to decrease at 1 h after adding myriocin, and the amounts of sphingomyelin in control and myriocin-treated cells at 72 h were 85.4 ± 0.6 and 25.8 ± 0.7 pmol/*μ*g protein, respectively. These results suggest that the present method of sphingomyelin quantification may reflect the myriocin-induced depletion of *de novo* sphingolipid synthesis. Under the same culture conditions, myriocin-treated cells revealed decreased growth and more floating cells compared to controls based on microscopic observation, indicating that sphingomyelin may be associated with cell growth and death (data not shown).

## 4. Discussion

Several methods have been developed to quantify sphingomyelin such as HPTLC, GC, NMR, and mass spectrometry. HPTLC can only quantify the relative intensity of sphingomyelin and is not possible to avoid the interfering compounds on TLC chromatogram [2]. For the GC analysis of sphingomyelin, the sensitivity and quality of analyses vary due to the high column temperature [3]. Plasma sphingomyelin from different animal species has been quantified by ^31^P NMR analysis [4]. MS analysis can quantify the molecular species of sphingomyelin with high sensitivity [5]. However, the sensitivity of MS analysis can be dependent on the mode of ionization and may vary with molecular species. The present analytical method can determine the sphingomyelin content without using either the relatively expensive instrument or the radioisotope. The present method includes HPTLC separation, enzyme hydrolysis, and HPLC analysis based on fluorescence detection. This analytical method for sphingomyelin quantification has several benefits compared with other methods. 

**Figure 3:**
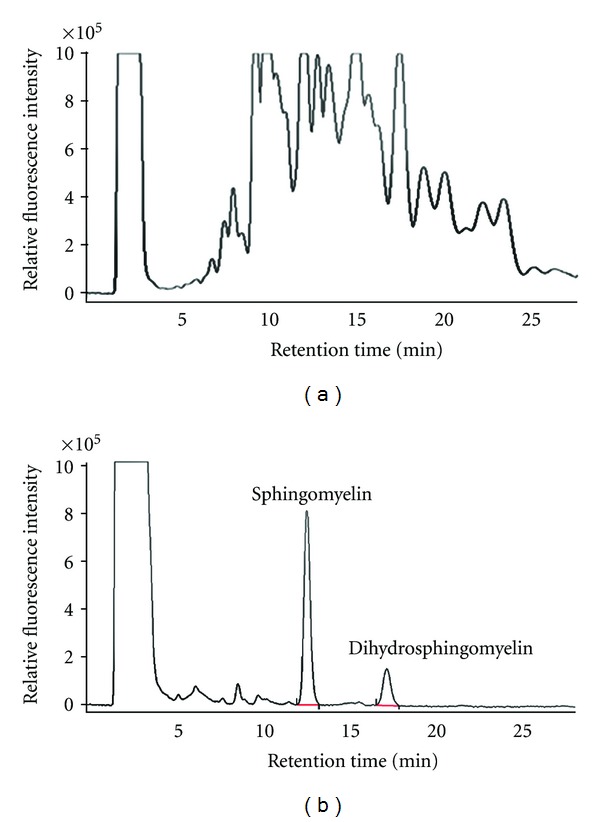
Effect of HPTLC separation for sphingomeylin on the background of HPLC chromatogram. Sphingomyelin of brain tissue from ICR mice was analyzed by HPLC (a) without and (b) with the process of HPTLC.

**Figure 4:**
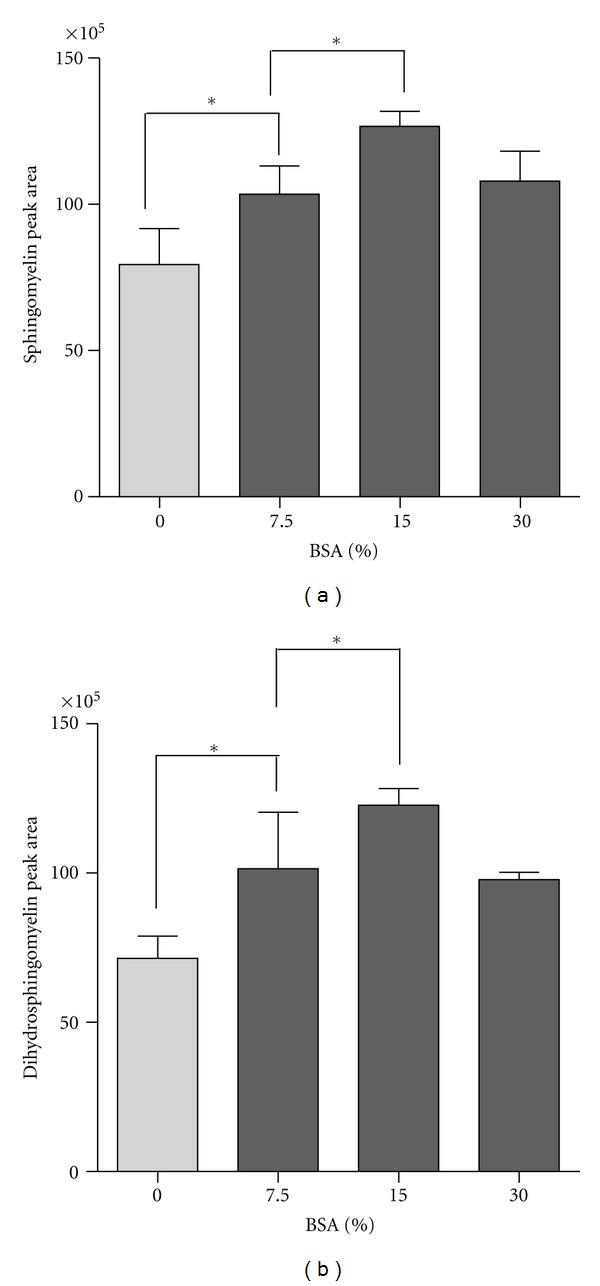
Effect of BSA on sphingomyelin hydrolysis. (a) Sphingomyelin and (b) dihydrosphingomyelin standards (1,000 pmol) were hydrolyzed with sphingolipid ceramide *N*-deacylase (SCDase) plus sphingomyelinase (SMase) in a reaction buffer with varying concentrations of fatty acid-free BSA (0, 7.5, 15, and 30%). The released sphingoid bases were derivatized with OPA and analyzed by HPLC. Values are expressed as the mean ± SD (*n* = 5). Differences with *P < 0.05 and **P < 0.01 were statistically significant.

**Figure 5:**
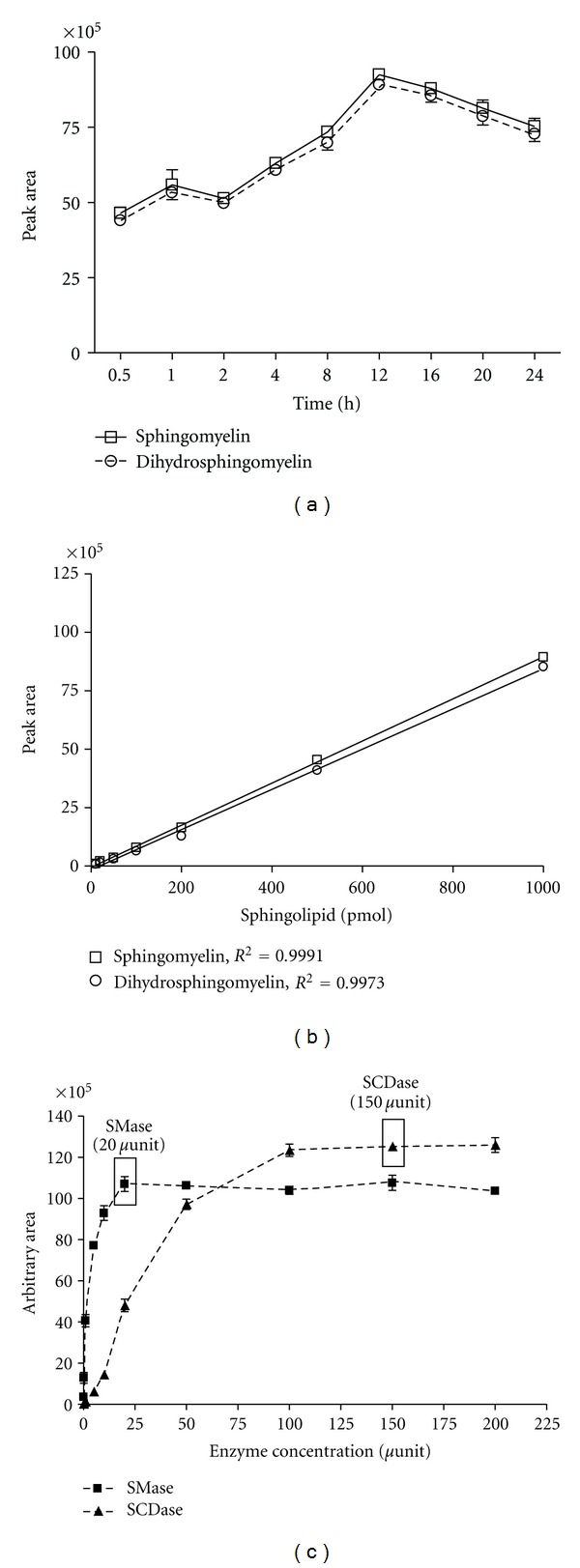
Optimization of enzymatic hydrolysis. Sphingomyelin and dihydrosphingomyelin standards were incubated (a) for varying periods of time (0.5, 1, 2, 4, 8, 12, 16, 20, and 24 h), (b) at the range of concentrations (10–1,000 pmol), and (c) 1,000 pmol sphingomyelin standard was simultaneously incubated with SMase and SCDase at the range of enzyme activities (5–200 *μ*units) for 12 h, followed by HPLC analysis. Values are expressed as the mean ± SD (*n* = 5).

**Figure 6:**
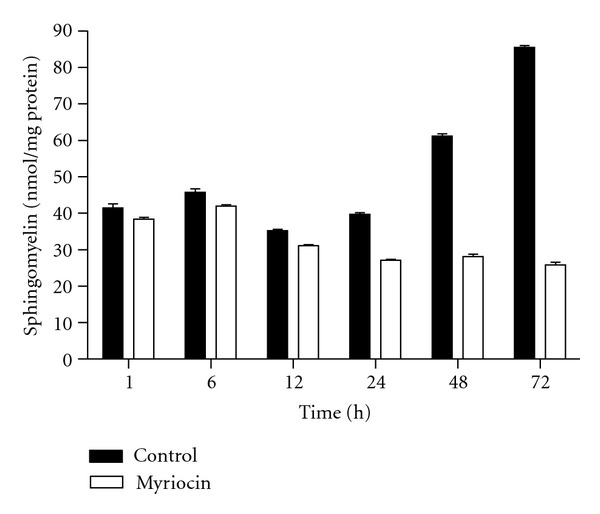
Inhibitory effect of myriocin on *de novo* sphingolipid synthesis. 3T3-L1 preadipocytes were grown for 48 h after seeding and then treated with 5 *μ*M myriocin for varying periods of time (1, 6, 12, 24, 48, and 72 h). Cellular concentrations of sphingomyelin ceramide were determined. Values are expressed as the mean ± SD (*n* = 3).

Spiking with dihydrosphingomyelin as an internal standard into biological samples makes the actual amounts of sphingomyelin possible to be measured and also compensated for variations and improved reproducibility. HPTLC separation of sphingomyelin excluded possible contamination with ceramide-1-phosphate, sphingosine-1-phosphate, ceramide, and sphingosine which may act as interfering lipids for sphingomyelin quantification, and also provided a clean background for HPLC, indicating that OPA fluorescence detection specific for the amino group of analytes requires the purification process (Figure 3).

Enzymatic hydrolysis and OPA fluorescence derivatization of amino group make this analytical method more specific and sensitive than previously published methods. Sphingomyelin and dihydrosphingomyelin of samples after HPTLC separation were specifically hydrolyzed with SCDase and SMase to produce sphingosine and dihydrosphingosine, respectively, which can be analyzed by HPLC. For the aspect of specificity, SMase cleaves the phophodiester bond of sphingomyelin yielding ceramide and phosphocholine, and SCDase hydrolyzes the *N*-acyl linkage between fatty acids and sphingoid bases in ceramides or sphingomyelin. The present HPLC method with OPA fluorescence detection can only detect the lipids with amino group. Thus, other endogenously occurring lipid compounds could not interfere with sphingomyelin analysis. 

Increasing sphingomyelin solubility for the enzyme reaction is the key to enhance the maximum hydrolysis of sphingomyelin in the present analytical method. Simultaneous hydrolysis of sphingomyelin with SCDase and SMase occurred efficiently with fatty acid-free BSA, because the formation of BSA-sphingomyelin complex appears to enhance sphingomyelin solubility.

Less variability and good recovery allow this analytical method to be applicable to diversity of biological samples for sphingomyelin quantification (Table 2), suggesting that spiking dihydrosphingomyelin as an internal standard into the biological samples may decrease the variation that could be caused by a multistep procedure.

Using fluorescence detection of HPLC systems allows LOQ to pmol levels, thus the amounts of samples equivalent to about 1 *μ*g protein or 5 *μ*L plasma are enough to determine sphingomyelin content of biological samples. Thus, the sensitivity of the present method was sufficient for analyzing sphingomyelin in small amounts of biological samples. The present method can be applied to a diversity of biological samples including mouse plasma and tissues (brain, kidney, and liver) and cultured cells (3T3-L1 cells, HT-29 cells, and RASMCs).

In conclusion, this analytical method is a sensitive and specific method for determining the total content of sphingomyelin in diverse biological samples and may be applicable to elucidating the molecular mechanisms of lipid-metabolism-associated diseases.

## Figures and Tables

**Table 1:** Intra- and inter-day CVs for the sphingomyelin standard.

Sphingomyelin			
	Added (pmol)	Measured (pmol)	CV^a^ (%)
Intra-day (*n* = 10)	100	98.18 ± 2.04	2.08
	200	189.05 ± 2.68	1.42
	500	483.68 ± 5.87	1.22
	1,000	980.51 ± 6.95	0.71

Inter-day (*n* = 10)	100	96.49 ± 2.14	2.22
	200	191.46 ± 2.55	1.33
	500	478.52 ± 5.57	1.16
	1,000	957.13 ± 6.68	0.70

^a^Coefficient of variation.

**Table 2:** Intra- and inter-day CVs and accuracy for the sphingomyelin in plasma and liver from ICR mice and 3T3-L1 cells.

Samples	Added	Intra-day (*n* = 10)			Inter-day (*n* = 10)		
		Measured^a^ (pmol)	CV^b^ (%)	Accuracy (%)	Measured^a^ (pmol)	CV^b^ (%)	Accuracy (%)
Mouse plasma (*μ*L)	1	405.51 ± 0.25	1.52	99.54	403.05 ± 0.20	1.17	98.93
	5	2004.22 ± 0.14	0.85	98.39	2018.23 ± 1.55	0.21	99.07
	10	3984.02 ± 3.15	0.80	97.80	4001.58 ± 6.50	2.50	98.21
	20	8320.05 ± 9.26	8.43	102.11	8247.09 ± 8.90	6.02	101.22

Mouse liver (*μ*g protein)	1	23.01 ± 0.02	0.71	103.37	22.05 ± 0.50	0.54	99.06
	2	44.50 ± 0.27	1.16	99.96	45.54 ± 2.15	1.27	102.30
	5	115.25 ± 3.90	0.98	103.55	109.46 ± 5.30	1.15	98.37
	10	219.19 ± 9.00	2.54	98.47	216.41 ± 7.25	1.72	97.22

3T3-L1 cells (*μ*g protein)	1	60.05 ± 0.15	0.10	99.92	62.00 ± 0.20	0.25	103.16
	2	121.47 ± 2.21	1.14	101.06	124.02 ± 5.44	2.10	103.18
	5	314.10 ± 8.06	1.21	104.53	308.95 ± 9.75	2.04	102.81
	10	587.12 ± 8.25	0.78	97.67	605.46 ± 6.15	1.45	100.74

^a^Values are expressed as the mean ± S.D. in *μ*M for mouse plasma and pmol/*μ*g protein for mouse tissues and cultured cells.

^b^Coefficient of variation.

**Table 3:** Levels of sphingomyelin in plasma and tissues from mice and cultured cells.

Source	Sphingomyelin^a^
ICR mice	
Plasma	407.40 ± 0.31
Brain	55.60 ± 0.43
Kidney	43.75 ± 0.21
Liver	22.26 ± 0.14
Cell type	
3T3-L1 cells	60.10 ± 0.24
HT-29 cells	58.38 ± 0.37
RASMCs	62.69 ± 0.08

^a^Values are expressed as the mean ± S.D. (*n* = 3) in *μ*M for mouse plasma and pmol/*μ*g protein for mouse tissues and cultured cells. RASMCs: rat aortic smooth muscle cells.
